# Needle Tip Tracking through Photoluminescence for Minimally Invasive Surgery

**DOI:** 10.3390/bios14100470

**Published:** 2024-09-30

**Authors:** Meenakshi Narayan, Mithun Bhowmick

**Affiliations:** 1 Department of Engineering Technology, Miami University, Middletown, OH 45042, USA; 2 Department of Mathematical and Physical Sciences, Miami University, Middletown, OH 45042, USA; bhowmim@miamioh.edu

**Keywords:** minimally invasive surgery, biosensors, photoluminescence, fluorescent markers, needle tip tracking

## Abstract

Minimally invasive surgery continues to prioritize patient safety by improving imaging techniques and tumor detection methods. In this work, an all-optical alternative to the current image based techniques for in vitro minimally invasive procedures has been explored. The technique uses a highly fluorescent marker for the surgical needle to be tracked inside simulated tissues. A series of markers were explored including inorganic (Perovskite and PbS) and organic (carbon dots) nanoparticles and organic dye (Rhodamine 6G) to identify layers of different stiffnesses within a tissue. Rhodamine 6G was chosen based on its high fluorescence signal to track 3D position of a surgical needle in a tissue. The needle was tracked inside homogeneous and inhomogeneous gelatin tissues successfully. This exploratory study of tissue characterization and needle tip tracking using fluorescent markers or photoluminescence technique show potential for real-time application of robot-assisted needle insertions during in vivo procedures.

## 1. Introduction

Minimally invasive surgery (MIS) offers benefits of increased accuracy and fast recovery for better patient outcomes since MIS uses small incisions to perform operations from outside the patient’s body. However, during needle-based interventions in soft tissues, MIS can face critical challenges of needle deflections and tissue deformations due to needle-tissue friction forces which can cause unexpected puncturing and bleeding [1]. For example, needle deflections and tissue deformations occurring due to dense layers of a biological tissue can steer away the needle from reaching desired target locations [2]. Because the surgeon controls the incision tool with limited visibility, having accurate information about the tissue environment is crucial to prevent these undesired events and ensure procedural safety [3,4]. Currently, many MIS procedures use either image-based or electromagnetic signal-based tracking techniques for positioning instruments inside the patient’s body [4]. Image-based techniques, such as ultrasound, face resolution issues, while magnetic resonance imaging (MRI) and computerized tomography (CT) scans are expensive and not real-time. X-ray imaging is harmful with prolonged use [4,5,6]. Electromagnetic tracking methods, on the other hand, are susceptible to noise and interference from nearby electromagnetic devices, requiring a combination of C-scan images for more accurate tissue environment mapping [7]. Recently, a combination of ultrasound and optoacoustic imaging have been explored in robot-assisted laproscopic procedures since this modality offers the cost-effective methods of ultrasound and high contrast of optical sensing [8]. This laser-generated ultrasound method improves contrast to detect needles within deep regions of the tissues. However, photoacoustic imaging technique also requires post processing segmentation techniques due to the low resolution of the ultrasound [9].

Over the years, several image based methods and control strategies such as tissue biomechanics models, path planners and novel needle designs were implemented to potentially reduce risks in MIS such as unwanted needle deflections and tissue deformations. However, biomechanics models require prior information of tissue properties, which is challenging for intra-operative procedures when tissue environments are unknown. Similarly, common imaging techniques [10,11,12], mechanical devices and probes as tissue constraints [13,14], and path planners [1,11,15] were implemented to control deformations of the tissue. Though these methods have shown success to achieve accurate target placement of the needle tip, they still rely on heavy finite element and biomehanics models for which mathematical modeling of tissues is necessary and is tough to obtain for heterogeneous tissues during intraoperative procedures. Moreover, accurate characterization of needle-tissue dynamics can be time consuming [16]. Recently, research groups are taking inspiration from parasitic invertebrates such as wasp ovipositor and mosquito, to design needles that can penetrate solid substrates easily without causing significant deformation to tissues [17,18]. These bio-inspired mechanisms of needle insertions can generally improve maneuverability, while reducing the aforementioned critical MIS events. However, a systematic study to select design parameters is required, which usually change due to varying heterogeneous tissue substrate [19]. Overall, these limitations highlight the need for alternative approaches that bypass the reliance on needle-tissue modeling and uncertain tissue environments. One such approach involves using fluorescent biomarkers to illuminate tissue and needle systems through laser excitation, coupled with data-driven control algorithms to prevent critical MIS events and enhance safety. As an initial step, this paper will focus on exploring fluorescent detection methods for characterizing tissues and tracking needle movement within the tissue.

Alternative methods such as fluorescence-based detection using Rhodamine 6G (R6G) dyes are currently being explored for their benefits of a simple portable setup, safety, and compatibility with microscopes. R6G is widely utilized in fluorescence microscopy, flow cytometry, and fluorescence correlation spectroscopy due to its high quantum yield, sensitivity, and strong contrast in visible light across various wavelengths [20]. This technique allows for the tracking of an object’s properties or densities by illuminating it with visible light after applying R6G, and then analyzing the fluorescent peaks through laser spectroscopy. Given that rhodamine is considered safe and biocompatible, this fluorescence-based approach holds significant potential for biomedical imaging and surgical applications [21]. Recently, similar illumination techniques have been employed to identify vital nerve structures during surgical procedures [22]. However, the use of fluorescence-based detection methods in needle-based interventions in soft tissues remains underexplored. Therefore, our work focuses on direct application of fluorescent or photoluminescence markers for needle tip tracking and tissue characterization. The idea is to test fluorescent materials as markers for the surgical needle.

Organic dyes are widely used as fluorescent tags in microscopy. However, in the past few decades, quantum materials were found to be more versatile with ample control options and extremely high quantum yields. Inorganic quantum dots are excellent candidates for their high quantum yields, room temperature stability and their versatile nature. Among them perovskite quantum dots are notable for their wavelength tunability and economic viability for a myriad of technological applications [23,24]. Perovskite quantum dots take a form of APbX_3_ (A = organic ammonium cation, X = halide ion). However, the main drawback for inorganic nanoparticles is their toxicity due to presence of metals such as Cd or Pb in them. A more recent development brought forward a novel solution to this: carbon dots (CD), which are greener, much less toxic options to traditional nanoparticles with high enough quantum yields to be useful for microscopy/sensing applications [25,26,27]. To our knowledge, there has not been any study comparing the traditional, inorganic quantum dots, more recently found highly fluorescent carbon dots, and organic dyes. Hence, this work focuses on novel detection methods using: (1) a representative organic dye such as R6G, (2) representative inorganic fluorescent nanoparticles such as Pb halide perovskite (PV) and lead sulphide (PbS), and (3) fluorescent CD. A comparison is made in terms of their respective responsiveness and suitability in sensing a static surgical needle through fluorescence. Strength of fluorescence signal was systematically probed to report on the sensitivity on spatial positions of the needle tip. Finally, the needle tip was tracked in 3D at various positions inside the gelatin sample. The results confirm traceability of the needle inside gelatin samples of all concentrations, and show strong sensitivity on the spatial position while inside the tissue. The proposed detection system shows potential for real-time needle tracking in robot-assisted surgeries.

## 2. Materials and Methods

This section outlines the procedure for preparing gelatin samples with varying stiffness levels and details the needle tracking methods using the photoluminescence setup.

### 2.1. Preparation of Gelatin Tissues and Needle Setup

**Figure 1:**
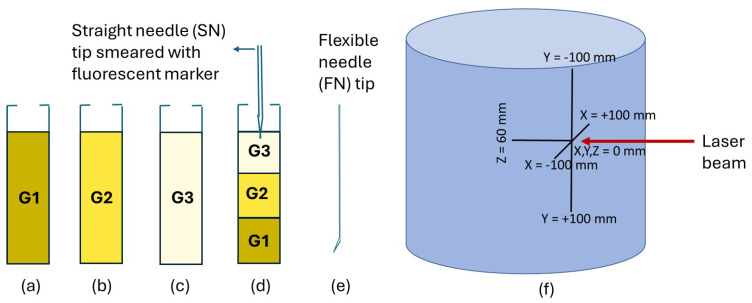
Needle-tip tracking in cuvettes with different density of gelatin samples. Three cuvettes containing homogeneous gelatin samples with highest stiffness (G1), medium stiffness (G2), and least stiffness of gelatin (G3) (**a**–**c**). Three-layered gelatin with a straight (SN) or a flexible (FN) needle tip smeared with fluorescent markers (**d**,**e**). 3D needle tip tracking in a gelatin sample (**f**).

A 1:3 ratio of gelatin to sugar with one teaspoon of gelatin was used for the samples. Pure cane granulated sugar (white) that is available in any grocery store was used. Similarly, Knox gelatin power derived from pork was used. Different densities or stiffness were achieved by varying the amount of water, as shown in Figure 1. For instance, one sample (G1) used 1 teaspoon of gelatin in 50 mL of boiling water. Another sample (G2) used 1 teaspoon of gelatin in 100 mL of boiling water. Third sample (G3) was prepared using 1 teaspoon of gelatin in 200 mL of boiling water. The liquid samples were poured into three cuvettes and refrigerated overnight, Figure 1a–c. To prepare the two-layered gelatin, one density (G1) was solidified in a cuvette. Then, the liquid sample of the other density (G2) was poured on top, and the mixture was refrigerated for 2 h. Next, the liquid mixture of G3 sample was poured onto the two-layered gelatin. The whole mixture was refrigerated overnight, forming a solid three-layered gelatin. The tips of the flexible needle (FN) and straight needle (SN) are smeared with fluorescent markers for tracking in different gelatin samples, as shown in Figure 1d,e.

### 2.2. Photoluminescent Markers

Both PbS and PV quantum dots were purchased from Milipore Sigma (St. Louis, MO, USA). The quantum dots came dispersed in toluene (10 mg/mL) and were used without further processing. The PbS nanoparticles were oleic acid coated with wavelength of emission at 900 nm, while the PV quantum dots were oleic acid and oleylamine coated with wavelength of emission at 530 nm. A dynamic light scattering (DLS) conducted in PbS and PV nanoparticle solutions determined the particle sizes to be 2.7 nm and 10 nm, respectively. CD nanoparticles with 450–700 nm emission were synthesized from ascorbic acid precursor through hydrothermal synthesis. A solution of ascorbic acid (1 g/25 mL) in deionized water, made from powder (Milipore Sigma, St. Louis, MO, USA) was put into an autoclave reactor at 180 degree Celsius for 24 h. The solution was then passed through a 25 μm filter to get rid of the larger sized particles, and the resulting solution was collected for using in the needle tracking experiments. DLS confirmed the carbon dot particle sizes to be 70 nm. R6G powder (dye content 99%) was acquired from Milipore Sigma (St. Louis, MO, USA) to make a solution in deionized water (1 g/25 mL), to be used in the needle tracking experiment.

### 2.3. 3D Needle Tip Tracking Using Luminescence

Needle-tip position tracking during needle insertions in a gelatin tissue sample is shown in Figure 1f. A laser beam shining along the Y-axis encounters the needle tip smeared with a luminescence marker such as R6G solution, to track the needle tip’s position within the gelatin tissue model. For example, the coordinates X-axis (0 mm to 200 mm), the Y-axis (0 mm to +200 mm), and the Z-axis (0 mm to 60 mm) show that the laser beam aligned along the Y-axis, provides a vertical reference line that helps in accurately determining the needle tip’s vertical position. By intersecting the needle tip, the laser beam enables precise measurement and tracking of the needle’s coordinates within the gelatin model, ensuring potential accurate placement and control necessary for experimental procedures.

### 2.4. Experimental Methods for Spectroscopy

**Figure 2:**
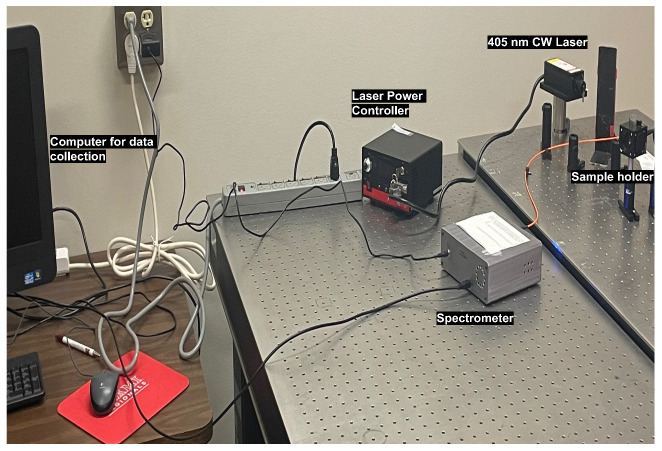
Experimental setup of the laser spectroscopy.

The needle tip coated with a luminescent solution such as R6G is inserted into a gelatin sample, leaving behind trail marks of R6G. After removing the needle tip, the gelatin stained with R6G is excited by a 405 nm collimated diode laser (Laser-glow Techonlogies, North York, ON, Canada). Four different luminescence markers including CD, PbS quantum dots, PV quantum dots, and R6G are used to compare the performance of fluorescence based tracking of needle tip in gelatin tissues. The fluorescence signal is collected using the homebuilt laser fluorescence set up comprising of a fiber-coupled spectrometer with a range 180–1100 nm (Silver Nova from StellerNet Inc., Tampa, FL, USA), custom-built cuvette holder with 450 nm long pass filter and a focusing lens, as well as a power meter (Laserglow Techonlogies, North York, ON, Canada.) for estimating fluence. The experimental setup is shown in Figure 2. The continuous wave (CW) laser intensities are tuned through a calibrated average power vs. diode current curve. Since it is critical to detect small signals from the tissue, average laser power is adjusted between 3.5 mW and 60 mW, both at the low end of the calibration curve in the linear region. It is also important to stay in the most sensitive region of the spectrometer calibration curve, which would be away from the saturation level of the detector inside the spectrometer.

## 3. Results and Discussion

**Figure 3:**
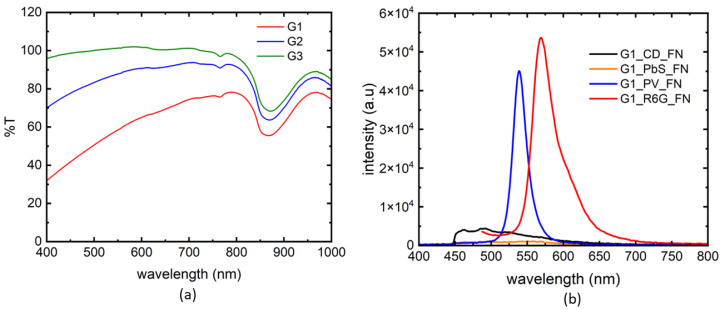
Comparison of fluorescent markers in terms of signal to noise ratio. Transmittance spectra for gelatin of different concentrations (**a**). A comparison of PL signal strength from four fluorescent markers, collected from the flexible needle (FN) tip (**b**).

Results of the transmission spectra for gelatin of different concentrations (G1 to G3) and intensity plots of the luminescence markers in G1 are shown in Figure 3.

There is no difference in %T for the three gelatin samples. We see fairly transparent spectra for all gelatin samples up to 800 nm. There is a dip at 850 nm wavelength, which could be a significant advantage if we wanted to use PbS nanoparticles. The loss of signal in %T could be due to the optical density differences, contributing to the opacity levels of the gelatin samples. Among the four markers tested, R6G showed strongest signal to noise, followed by PV nanoparticles. PbS PL was the weakest and it is not clear if there is any significant contribution from gelatin absorption at around 850 nm. CD showed stronger, but extremely broad PL signal compared to PbS nanoparticles.

**Figure 4:**
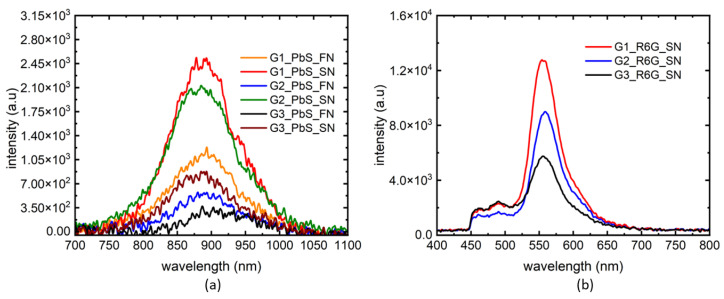
Effect of needle types (FN, SN) on the performance of fluorescent markers. Comparison of PL signals from FN and SN through 3 different concentrations of gelatin samples using PbS marker (**a**) and R6G marker (**b**).

The intensity plots of the absorption spectra for three gelatin concentrations using PbS and R6G markers are shown in Figure 4. Both FN and SN needles were used to study the effect of needle types. Even the weakest photoluminescence (PL) signal is capable of tracing a needle through all 3 concentrations of gelatin samples according to Figure 4a. We noted that the PL from SN was always higher than that of FN in the gelatin samples. This could be due to the design of the FN vs. SN, where SN has a wider diameter to house more luminescence marker, compared to the prebent tip of the FN with a lower tip surface area. However, the signal from FN was still significant to identify different gelatin layers. We chose SN for subsequent needle tracking experiments with the most fluorescent marker R6G. From Figure 4b, we note that the PL signal was highest for the most dense gelatin layer (G1), and lowest for the least dense gelatin layer (G3). This shows that different layers of tissue could be mapped using the peak values of the PL signals. For future work, we will validate this method of tissue characterization using a heterogeneous (biological ex vivo) tissue using the PL intensities of the absorption spectra. Also, the emission peak for R6G is at 560 nm, which is away from the absorption dip seen in the %T of Figure 3 at 850 nm. We note that FNs may have a lot of potentials if the PL signal is optimized for an all-optical sensing technique.

**Figure 5:**
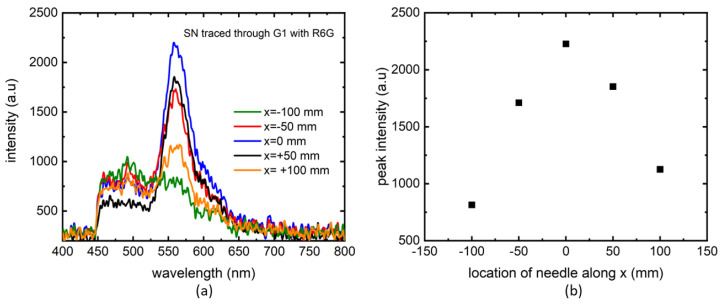
First attempt to track a needle through G1 using R6G as marker along x-axis. The PL measurements are plotted for different locations of the needle with respect to the excitation laser (**a**). PL peak intensities at those locations (**b**).

**Figure 6:**
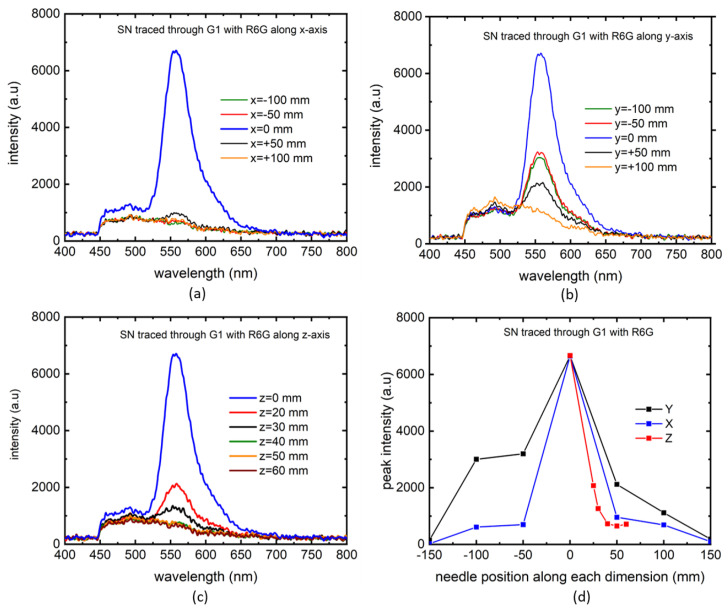
Tracking of SN through G1 with R6G as marker along x (**a**), y (**b**), z (**c**) directions. PL peak intensities as functions of needle positions along the 3 dimensions (**d**).

In the first trial, we started the needle tracing from the location of maximum PL intensity along x-axis, meaning, we maximized the PL signal on the spectrometer by adjusting the laser along x-axis and then moved away along x-axis on both sides to check the PL intensity drop with y- and z- coordinates unchanged, see Figure 1f. No optimization adjustments were made for the y- and z-axis for this trial. The resulting traces are seen in Figure 5. The PL traces were sensitive to the needle position, confirming our approach. In this trial the highest dense gelatin (G1) was used since we noted maximum possible PL signal to noise ratio in that sample. Since the needle position is already “known” through maximum PL intensity alignment, we could collect data for gradual intensity loss. To probe the viability of finding the signal when the needle is not aligned to the maximum signal location, a second set of experiments were performed, presented in Figure 6.

In a more general approach, the needle (SN) positions were tracked through G1 in all dimensions. In this approach, we started far away from the optimized PL signal position of the needle, and gradually scanned the container along one axis at a time. As can be seen in Figure 6a, the experiment started with no visible PL signal and then drastically increased to maximum as the optimal position is reached. This evidences the sensitivity of the technique and its potential as a precise detector of a surgical needle. A similar approach was taken along the y-axis, producing similar sensitivity (Figure 6b). Along z-axis, the experiment was performed in a slightly different way since this is the dimension where the excitation laser was aligned to, with the results shown in Figure 6c. To determine the maximum depth along z-axis where some signal could be found, we started from the most optimized location and gradually moved away from the excitation source. There was a hint of signal at 40 mm, marking the extreme coordinate for the z-axis. Finally, a summary of these experiments are presented in Figure 6d, showing a virtual map of the needle tip constructed by overlapping PL peak intensities in all dimensions. For consistency and to avoid confusion, the same PL measurement data was used in Figure 6a–c for the maximum intensity PL marking the x=y=z=0 position.

**Figure 7:**
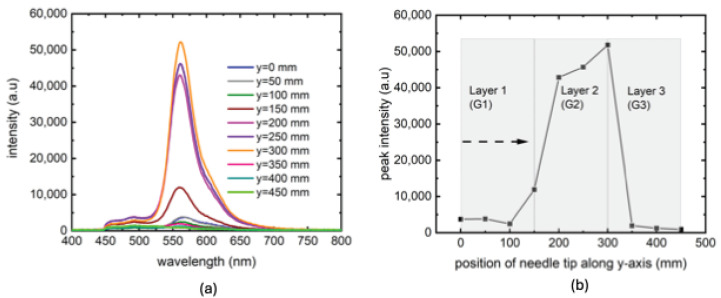
Tracking of SN through a 3-layered gelatin sample along y-axis (**a**). PL peak intensities as a function of position along y-axis with the dashed arrow showing the direction of scan (**b**).

To investigate the success of this technique in a more nonuniform sample, a series of experiments were performed in a gelatin sample with 3 different layers of concentrations, presented in Figure 7. In this experiment, we traced the needle tip along y-axis, the dimension along which gelatin concentrations were different. The resulting PL traces are shown in Figure 7a. Unlike previous labeling schemes, this experiment uses a gradual increase of needle tip position from 0 (edge of the first gelatin layer at the top of the cuvette where first detectable PL signal was found) to 450 mm (the farthest position in the bottom layer where PL signal became undetectable). As could be seen in Figure 7b, the PL peak signals showed strong sensitivity with the needle tip positions, especially in the middle layer where the excitation laser was aligned. These results show that the technique works at least with same efficiency as in single layer gelatin experiments. It is possible that the relative intensities of PL peaks could also be used to detect tissue densities, a prospect we would like to pursue in our future work using ex-vivo biological tissues with higher degrees of inhomogeneity. Another interesting prospect could be to design a probe with portable excitation laser of low power and scan a tissue with it, while keeping the needle in one location.

For future real time tracking of needle tip, the excitation laser source could be positioned onto the robot end-effector in the perpendicular direction to the surface of the tissue from which the needle is inserted, such that PL signal changes with the moving needle in a normal distribution and needle-tip position could be obtained from this relationship. Future work will compare our photoluminescence methods of tissue characterization and needle tip tracking with existing photoacoustic and ultrasound imaging methods [28].

## 4. Conclusions

This paper introduced an approach for tracking the 3D position of a needle tip within homogeneous and nonhomogeneous tissues using photoluminescence or fluorescence techniques. We were also able to detect peak intensities relative to different gelatin layers that could be used to characterize tissue environments. Four fluorescent markers—R6G, carbon dots, and inorganic dyes (PbS and Perovskite)—were evaluated to identify the most effective in detecting the needle. While all four markers were capable of distinguishing between different gelatin layers, R6G demonstrated superior contrast. The study involved the insertion of both straight and flexible needles coated with fluorescent markers into gelatin samples to assess the impact of needle type on photoluminescence. Due to the design limitations of the flexible needle, the straight needle produced a stronger photoluminescent signal. Overall, the straight needle coated with R6G provided the best contrast for detecting tissue layers and the needle tip.

The focus of this paper was mainly to check viability of the photoluminescence method for needle tracking in homogeneous tissues with known environments such as gelatin. Future research will aim to validate the proposed photoluminescence detection methods in conjunction with photoacoustic imaging to track needles in biological tissues. Additionally, algorithms will be developed to map photoluminescent signals with tissue environments and needle-tip positions, complemented by data-driven control techniques to accurately guide the needle tip to target locations during needle interventions in real time.

## Data Availability

The data presented in this study are available on request from the corresponding author due to university policies restricting public access to data.
